# Supplementary material for: Tumorigenicity decrease in Bcl-xL deficient MDCK cells ensuring the safety for influenza vaccine production
Source: PLoS One. 2024 Dec 16;19(12):e0311069. doi: 10.1371/journal.pone.0311069 (PMC11649150; doi:10.1371/journal.pone.0311069)
Supplement: S1 File — (ZIP) [file pone.0311069.s002.zip › S2_File/3_KEGG_enrichment/scatter/B_vs_A.sign_KEGG_enrichment_scatter.pdf]

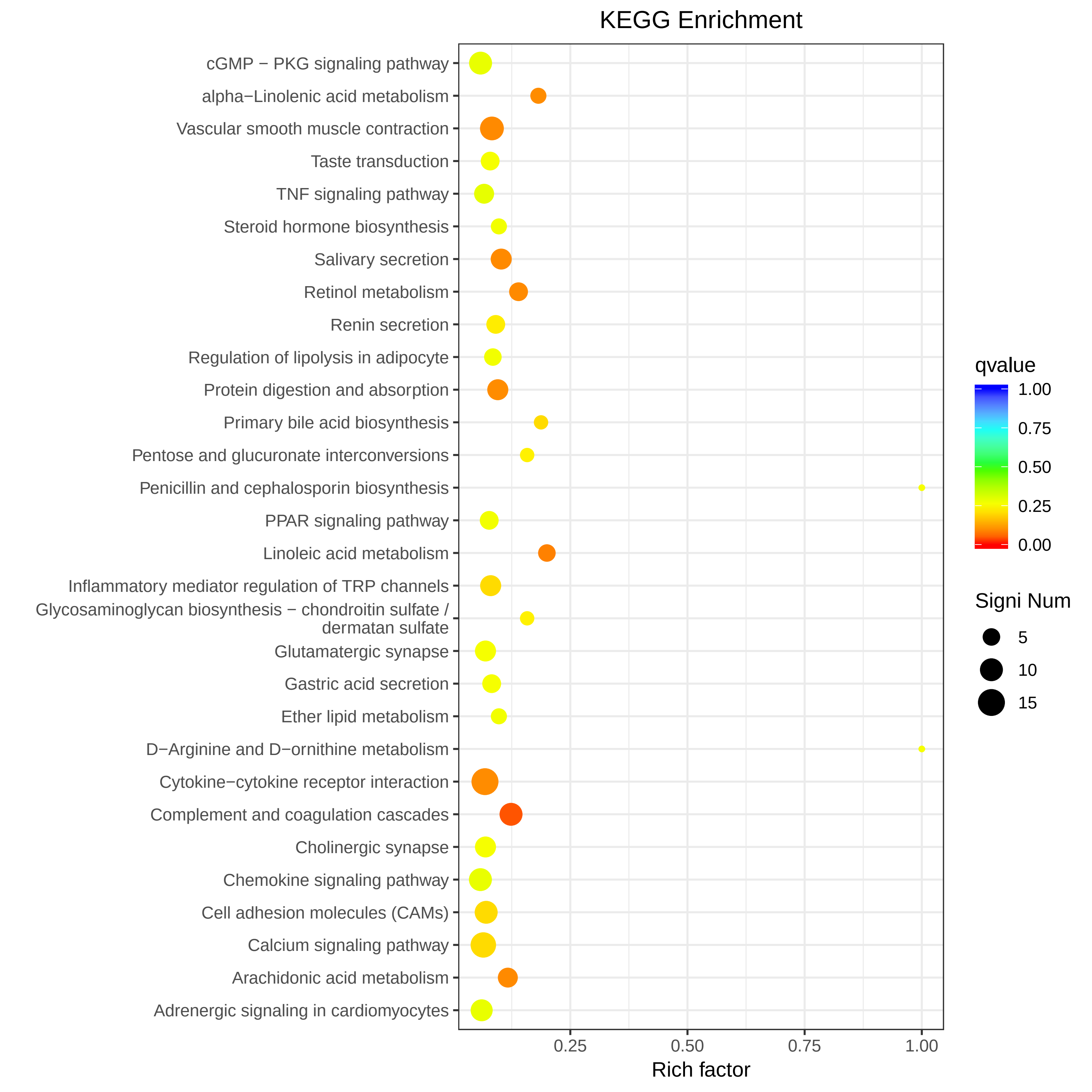

KEGG Enrichment
cGMP − PKG signaling pathway
alpha−Linolenic acid metabolism
Vascular smooth muscle contraction
Taste transduction
TNF signaling pathway
Steroid hormone biosynthesis
Salivary secretion
Retinol metabolism
Renin secretion
Regulation of lipolysis in adipocyte
Protein digestion and absorption
Primary bile acid biosynthesis
Pentose and glucuronate interconversions
Penicillin and cephalosporin biosynthesis
PPAR signaling pathway
Linoleic acid metabolism
Inflammatory mediator regulation of TRP channels
Glycosaminoglycan biosynthesis − chondroitin sulfate / dermatan sulfate
Glutamatergic synapse
Gastric acid secretion
Ether lipid metabolism
D−Arginine and D−ornithine metabolism
Cytokine−cytokine receptor interaction
Complement and coagulation cascades
Cholinergic synapse
Chemokine signaling pathway
Cell adhesion molecules (CAMs)
Calcium signaling pathway
Arachidonic acid metabolism
Adrenergic signaling in cardiomyocytes
0.25
0.50
0.75
1.00
Rich factor
qvalue
1.00
0.75
0.50
0.25
0.00
Signi Num
5
10
15
